# Low probability of myasthenia Gravis in patients presenting to neuro-ophthalmology clinic for evaluation of isolated ptosis

**DOI:** 10.1177/11206721221107300

**Published:** 2022-06-09

**Authors:** Laura Donaldson, Mariam Issa, Victoria Dezard, Edward Margolin

**Affiliations:** 1 Department of Surgery, Division of Ophthalmology, 3710 McMaster University, Hamilton, Ontario, Canada; 2 Faculty of Medicine, 7938 University of Toronto, Toronto, Ontario, Canada; 3 Department of Ophthalmology and Vision Sciences, Faculty of Medicine, 7938 University of Toronto, Toronto, Ontario, Canada; 4 Department of Medicine, Division of Neurology, Faculty of Medicine, 7938 University of Toronto, Toronto, Ontario, Canada

**Keywords:** myasthenia gravis, ptosis, diplopia, sensitivity

## Abstract

**Background:**

Concerning causes of ptosis, most notably third nerve palsy and Horner's syndrome, can be ruled out with normal ocular motility and pupillary examination. Myasthenia gravis (MG) however, rarely can present with ptosis as an isolated finding. We reviewed all patients presenting to tertiary neuro-ophthalmology practice with ptosis of unknown etiology to determine the frequency of MG.

**Methods:**

Retrospective chart review of patients referred to a tertiary neuro-ophthalmology practice with undifferentiated ptosis.

**Results:**

Sixty patients were included in the study. Twenty eight (47%) patients had ptosis along with various abnormalities of ocular motility and/or alignment and 32 (53%) had isolated unilateral ptosis defined as ptosis with absence of diplopia, or symptoms of generalized MG (GMG). Final diagnosis was aponeurotic ptosis due to levator palpebrae dehiscence in the majority (73%) of patients, while 10 (17%) were diagnosed with MG (6 with OMG, 4 with GMG). Diplopia was present in 9/10 patients with MG and 8/10 had abnormal ocular findings on clinical examination such as orbicularis oculi weakness, Cogan's lid twitch or fatiguability of ptosis on sustained upgaze. Only one patient referred for isolated unilateral ptosis was diagnosed with OMG and this patient had orbicularis oculi weakness.

**Conclusions:**

None of the patients with isolated unilateral ptosis and otherwise normal examination had MG. All patients eventually diagnosed with MG had diplopia or orbicularis weakness on examination. Thus, the yield of investigating patients with isolated ptosis for MG is exceedingly low.

## Introduction

Acquired blepharoptosis has a broad differential diagnosis, including aponeurotic, myogenic, neurogenic and neuromuscular conditions.^1^ Most patients with ptosis will have benign underlying etiology with age-related gradual dehiscence of the levator palpebrae aponeurosis (aponeurotic ptosis) being the most common cause. Aponeurosis dehiscence is characterized by a high upper lid crease with normal levator palpebrae superioris function.^2^ However, some patient with ptosis may harbor sinister pathology and it is important to differentiate benign aponeurotic ptosis from other potentially life-threatening conditions such as third nerve palsy, Horner's syndrome or myasthenia gravis (MG).

Careful clinical examination is the key to ruling out these concerning causes of ptosis. It is exceedingly rare for third nerve palsy to present with isolated ptosis as ocular motility is almost always abnormal in this condition and pupil mydriasis is often present. Horner's syndrome is associated with miotic pupil andPresence can be confirmed by pupillary responses to specific pharmacologic agents.^3^

MG is a systemic autoimmune disorder affecting the neuromuscular junction leading to skeletal muscle weakness characterized by fatiguability and fluctuating symptoms. Extraocular muscles and levator palpebrae are the most commonly affected muscles and ptosis and diplopia are often the initial presentation of MG.s. Although some patients will go on to develop generalized disease (generalized MG; GMG), 25–50%% of patients will have isolated ocular myasthenia gravis (OMG).^4–7^ The prevalence of MG in patients who present with isolated ptosis is unknown. One small series reported 6% of patients with undifferentiated ptosis eventually being diagnosed with MG, however, it is not clear whether these patients also had other symptoms or signs.^8^

Diagnosis of OMG can be challenging to arrive at especially after a single encounter when the variable nature of ptosis and ocular motility is not evident and specific investigations for MG are routinely required Serologic testing for acetylcholine receptor antibodies (AChRAbs) has greater than 95% specificity and the sensitivity in GMG is 80–90%. In OMG however, sensitivity is only around 50–60%.^9–11^ Single fiber EMG (sfEMG) is the most sensitive test for MG at 80–100% but does depend on the expertise of the examiner.^10,12–14^ Other testing modalities include cholinesterase inhibitor challenge, ice pack testing and fatiguability on sustained upgaze.

We reviewed the charts of patients referred to tertiary neuro-ophthalmology practice for undifferentiated ptosis. Their presenting clinical features were correlated with the final diagnosis in order to establish a frequency of MG in this patient population to help clinicians in guiding investigations for patients presenting with ptosis.

## Methods

A retrospective chart review of patients over 18 years of age presenting to tertiary centre neuro-ophthalmology practice between 2017 and 2021 with unclassified ptosis was conducted. Patients referred for a known diagnosis of MG or with other causes of ptosis such as third nerve palsy and Horner's syndrome were not included. The study was approved by the Health Sciences Research Ethics Board at the University of Toronto and adhered to the tenets of the Declaration of Helsinki.

Data collected included patient demographics (age, gender, presence of other autoimmune disorders) referral patterns (referring physician, reason for referral), presenting clinical features (ptosis onset, laterality, fluctuation and fatiguability, presence of diplopia, shortness of breath (SOB), muscle weakness, voice changes, dysphagia) and clinical signs (pupillary abnormalities, ocular motility, orbicularis weakness, Cogan's lid twitch, fatiguability on sustained upgaze and proximal limb muscle weakness). Final diagnosis of MG was based on clinical features along with either detectable AChRAb) or abnormal sfEMG, as well as evaluation by a neuromuscular neurologist who concurred with the diagnosis. The ice pack test and edrophonium testing were not routinely used. AChRAbs were ordered for all patients without an alternate etiology of ptosis evident after their first examination. Patients were excluded if they did not complete serologic testing was ordered but not completed or if they did not attend a follow-up appointment.

Results are presented as sensitivity and specificity values with respective 95% confidence intervals (CIs). Univariant analysis, including *t* test and Fisher Exact test, was used when appropriate. Statistical analysis was preformed using SPSS (Version 28.0, IBM Corporation).

## Results

Sixty six patients with undifferentiation ptosis were identified and of these, 6 were excluded due to lack of follow-up and results of serologic testing for a total of 60 patients included in the study. Ptosis was unilateral in 40 (66.7%). The mean age at presentation was 56.3 ± 18.8 years (range: 13 to 87 years) and 55% (33/60) of the patients were women (Table 1). Most patients were referred by ophthalmologists (45/60, 75%), followed by optometrists or family physicians (6/60, 10% each), and neurologists (3/60, 5%). MG was suspected in 50% (30/60) of patients by the referring physicians, 93.3% (28/30) of whom were ophthalmologists. AChRAbs were ordered in 48/60 patients (80%) where no clearetiology of ptosis was evident after the initial clinical assessment (11 patients with obvious aponeurotic ptosis and 1 with chronic progressive external ophthalmoplegia [CPEO]). Thirty patients (53%) had isolated unilateral ptosis, defined as ptosis in the absence of any symptoms of MG (diplopia, shortness of breath, voice changes, dysphagia, muscle weakness).

**Table 1.** Demographics of patients referred for evaluation of ptosis.

Demographics		N of patients (%) N = 60
Age at presentation	56.3 ± 18.8	
Female (%)		33 (55)
Referring physician	Ophthalmologist	45 (75)
	Optometrist	6 (10)
	Neurologist	3 (5)
	Family Physician	6 (10)
Reason for referral	Query MG	30 (50)
	Ptosis	18 (30)
	Diplopia	4 (6.7)
	Other	8 (13.3)

Final diagnoses are presented in Table 2. Aponeurotic ptosis was the most common cause of ptosis, diagnosed in 73% of all patients (44/60). MG was eventually diagnosed in 17% (10/60) it was confined to the ocular muscles in 60% (6/10) and generalized in 40% (4/10). Neurogenic contributors for ptosis included early-onset Parkinson's disease (1/60, 1.7%) and Horner's syndrome (1/60, 1.7%). Myogenic contributors included CPEO (2/60, 3.3%) and orbital myositis (1/60, 1.7%).

**Table 2.** Final diagnosis for patients presenting for evaluation of ptosis.

Final diagnosis			Unilateral	Bilateral	N of patients (%) (N = 60)
MG	Seropositive		5	2	7 (11.7)
	Seronegative		1	1	2 (3.3)
	Not available		1	0	1 (1.7)
Non-MG	Neurogenic	Parkinson's disease	1	0	1 (1.7)
		Horner's syndrome	0	1	1 (1.7)
	Myogenic	CPEO	0	2	2 (3.3)
		Myositis	1	0	1 (1.7)
	Aponeurotic ptosis		30	14	44 (73.3)
	Congenital		1	0	1 (1.7)

CPEO: Chronic progressive external ophthalmoplegia.

MG: Myasthenia gravis.

There was no statistically significant difference in the mean age at presentation between the MG and non-MG cohorts (65.5 ± 15.6 years versus 54.4 ± 19.0, respectively, Table 3). The proportion of females was also not significantly different between the two groups (60% versus 54%). Of the 10 patients eventually diagnosed with MG, 6 were suspected to have MG by the referring physician, 2 were referred for isolated ptosis and 2 for diplopia. Referral by any physician for suspected MG had a sensitivity of 60% (CI; 26–88%), and specificity of 52% (CI; 37–66%) for the diagnosis, whereas referrals by ophthalmologists had a sensitivity of 67% (CI; 23–96%) and specificity of 39% (CI: 23–55%). None of the patients in the MG group had another known autoimmune disease.

**Table 3.** Clinical features in patients presenting for evaluation of ptosis.

Variable		Total (N = 60)	MG (N = 10)	Aponeurotic (N = 44)	Other (N = 6)	P value (MG vs. Non-MG)
Age at presentation		56.3 ± 18.8	65.5 ± 15.6	54.2 ± 18.4	56.0 ± 24.9	0.80
Referral	Query MG	30 (50)	6 (60)	21	3	0.73
Time since ptosis onset	≤ 3 months	15 (25)	5 (50)	9	1	0.10
	4–12 months	11 (18.3)	4 (40)	7	0	0.07
	13–35 months	13 (21.7)	0 (0)	11	2	0.10
	≥ 36 months	15 (25)	1 (10)	11	3	0.43
	Not available	6 (10)	-	6	0	
Clinical symptoms	Fatigable	32/58 (55.2)	8 (80)	22	2/4	0.16
	Fluctuate	22/59 (37.3)	5 (50)	13	4/5	0.48
	Diplopia	22/59 (37.3)	9 (90)	10	3	**0.0003**
	SOB	7/59 (11.9)	0 (0)	5	2/5	0.59
	Muscle weakness	5/59 (8.5)	1 (10)	2	2/5	1.00
	Voice changes	2/58 (3.4)	0 (0)	1	1/4	1.00
	Dysphagia	6/58 (10.3)	0 (0)	5	1/4	0.58
	Unilateral ptosis	41 (68.3)	7 (70)	30	4	0.32
	Bilateral ptosis	19 (31.7)	3 (30)	14	2	0.30
	Isolated unilateral ptosis	32 (53)	1 (10)	29	2	**0.004**
Clinical signs	Anisocoria, smaller pupil on ptotic side	4/52 (7.7)	1 (10)	3/37	0/5	1.00
	Larger pupil on ptotic side	3/52 (5.8)	0 (0)	2/37	1/5	1.00
	Abnormal EOMs	10 (16.7)	3 (30)	4	3	0.35
	Orbicularis weakness	26/59 (44)	8 (80)	17 /43	2/6	**0.0163**
	Cogan lid twitch positive	7/44 (15.9)	3/6 (50)	3 /32	0/6	**0.0419**
	Fatiguability on sustained upgaze	11/45 (24.4)	5/7 (71.4)	6/35	0/3	**0.0061**
	Muscle weakness	11/56 (19.6)	3/9 (33.3)	6/42	2/5	0.36
Abnormal diagnostic tests	AChRAb	7/44 (15.9)	7/9 (77.7)	0/30	0/5 (0)	**0.00001**

MG: myasthenia gravis.

SOB: shortness of breath.

AChRAb: acetylcholine receptor binding antibody.

In the MG cohort, diplopia was present in 9 (90%), worsening of ptosis at the end of the day in 8 (80%) and fluctuation of ptosis in 5 (50%). Only one MG patient (10%) endorsed muscle weakness on history, though 33.3% (3/9) were found to have proximal muscle weakness on physical exam and were subsequently diagnosed with GMG. Diplopia was significantly more prevalent in the MG compared to the non-MG cohort (MG: 90%, Non-MG: 26.5%, P = 0.0003), whereas isolated unilateral ptosis was significantly more prevalent in the non-MG cohort (MG: 10%, Non-MG: 62%, P = 0.004). Only one MG patient (10%) presented with isolated unilateral ptosis, however, this patient had abnormal orbicularis strength on examination.

At least one ocular test performed in clinic (abnormal ocular motility, orbicularis oculi weakness, Cogan's lid twitch, fatiguability on sustained upgaze) was abnormal in 80% (8/10) of MG patients. Orbicularis oculi muscle weakness, Cogan's lid twitch and fatiguability on sustained upgaze were significantly more prevalent in the MG compared to non-MG cohort [(orbicularis oculi muscle weakness; MG = 80%, non-MG = 36.7%, p = 0.0163), (Cogan's lid twitch; MG = 50%, non-MG = 10.5%, p = 0.0419), (fatiguability on sustained upgaze; MG = 71.4%, non-MG = 15.8%, p = 0.0061)]. AChRAb testing was performed in 44 patients (OMG: 6, GMG: 4, Non-MG: 35), and the antibodies were detected in 70% (7/10) of MG patients; 50% (3/6) of patients with OMG and 100% (4/4) of patients with GMG. All seronegative OMG patients had orbicularis oculi muscle weakness, abnormal ocular alignment and fatigable symptoms and had abnormal sfEMG. They were all evaluated by a neuromuscular neurologist who concurred with the diagnosis.

Sensitivity and specificity of varying clinical findings for the diagnosis of MG are listed in Table 4. Notably, unilateral ptosis without any MG symptoms had a sensitivity of 62% (47–75%) and a specificity of 90% (56–100%) for a non-MG etiology (Table 4) and presence of diplopia had a sensitivity of 90% (CI; 55–99%) and a specificity of 73% (CI; 60–85%) for the diagnosis of MG.

**Table 4.** Sensitivity and specificity of clinical and serologic tests for myasthenia gravis.

Clinical feature / diagnostic test	Sensitivity % (95% CI)	Specificity % (95% CI)
Diplopia	90 (55–99)	73 (60–85)
Orbicularis oculi weakness	80 (44–97)	63 (48–77)
Fatiguability on sustained upgaze	71 (29–96)	84 (69–94)
Muscle weakness	33 (8–70)	83 (69–92)
Cogan lid twitch positive	50 (12–88)	89 (75–97)
Abnormal EOMs	30 (7– 65)	86 (73–94)
AChRAb positive	78 (40–97)	100 (90–100)
Non-MG diagnosis		
Isolated unilateral ptosis	90 (56-100)*	62 (47-75)*

AChRAb: acetylcholine receptor binding antibody.

## Discussion

Despite a high number of referrals for suspected MG, the majority of patients in our series (75%) presenting for evaluation of ptosis were found to have benign etiology of aponeurotic ptosis.

In the evaluation of patients referred for ptosis, a thorough history and careful ocular examination which includes pupillary examination, ocular motility and alignment testing is key. Diplopia and/or ptosis are the most common presenting symptoms in up to 85% of MG patients^15,16^ and are the only symptoms present at MG onset in up to 50%.^16^ In our series, 9/10 MG patients who presented with ptosis also had diplopia and isolated unilateral ptosis had very high specificity (90%) for non-MG diagnosis.

Notably in both our MG and non-MG cohorts, more patients reported diplopia than were found to have abnormal ocular motility on examination. Due to the intermittent nature of MG symptoms, an obvious motility defect may not be present at any given time. In addition, diplopia may be reported in non-MG patients with benign entities such as decompensated phorias or monocular diplopia may be present thus diplopia has a relatively poor specificity for MG, 73% in our cohort.

Only a single patient eventually diagnosed with seropositive OMG presented with isolated unilateral ptosis. While we defined isolated ptosis based on absence of symptoms of MG (e.g. diplopia, bulbar symptoms and proximal muscle weakness), this patient did have orbicularis oculi weakness on examination, emphasizing that while absence of any of the symptoms of MG is a sensitive approach for ruling out this diagnosis in patients with ptosis, abnormalities on clinical examination increase this sensitivity even further. At least one of the bedside ocular tests (abnormal EOM, orbicularis oculi weakness, Cogan's lid twitch, fatigability on sustained up gaze or muscle weakness) was abnormal in 80% of MG patients. Assessment of orbicularis oculi muscle strength and for presence of ptosis fatiguability on sustained upgaze are simple and efficient bedside tests that had a sensitivities of 80% and 71% in our series for identifying patients with MG.

AChRAbs were positive in all 4 cases of GMG and 3/6 cases (50%) of OMG, which is consistent with the published literature.^17–19^ Seropositivity in OMG has been associated with duration of MG symptoms, however, variable symptom onset has been found in seronegative patients.^20^ 3 seronegative OMG cases in our series had duration of ptosis of 2 weeks, 1 month and 17 years suggesting that serum AChRAb levels are either never high enough to detect in some patients, or there are antibodies that are unknown/undetectable due to limitations in current techniques.

This study has several limitations, most importantly its retrospective nature. Some patients did not complete serologic testing for AChRAbs but were classified as non-MG due to the low index of suspicion for MG after detailed history and neuro-ophthalmologic examination and lack of concerning findings or fluctuating signs and symptoms on follow-up examinations. In addition, our sample size was relatively small. Our cohort was seen at a tertiary centre by an experienced neuro-ophthalmologist We did not include patients with other causes of ptosis, however, clinicians with less experience in evaluation of diplopia may not as readily recognize and exclude these cases, particularly subtle third nerve palsies.

## Conclusions

The underlying condition in most patients with undifferentiated ptosis is levator aponeurosis dehiscence. Absence of all MG symptoms is very sensitive for ruling out the diagnosis. In patients with isolated ptosis, absence of MG symptoms (diplopia, bulbar symptoms and proximal muscle weakness) and normal ocular motility, alignment and orbicularis strength on examination essentially rule out the diagnosis of MG obviating the need for further testing.
